# Effect of Illite Treatment on Quality Characteristics and Antioxidant Activity of Broccoli (*Brassica oleracea* L. var. *italica*) Sprouts

**DOI:** 10.3390/molecules29184347

**Published:** 2024-09-13

**Authors:** So-Hyun Kim, Sanjeev Kumar Dhungana, Il-Doo Kim, Arjun Adhikari, Jeong-Ho Kim

**Affiliations:** 1 Department of Applied Biosciences, Kyungpook National University, Daegu 41556, Republic of Korea; gus5288@naver.com (S.-H.K.); arjun@knu.ac.kr (A.A.); 2 Division of Agriculture and Natural Resources, University of California, Exeter, CA 93221, USA; skdhungana@ucanr.edu; 3 International Institute of Research and Development, Kyungpook National University, Daegu 41556, Republic of Korea; ildookim@hanmail.net; 4 Department of Green Technology Convergence, Konkuk University, Chungju 27478, Republic of Korea

**Keywords:** microgreen, nutrition, sulforaphane, compounds, mineral elements, antioxidant

## Abstract

Microgreens have recently gained popularity owing to their reliable economic and nutritional value. This study aimed to increase the quality of microgreen broccoli via treatment with different concentrations (1%, IPB-1; 3%, IPB-3; 5%, IPB-5; or 7%, IPB-7 *w*/*v*) of illite—a natural mineral powder. The results showed that the illite treatments considerably increased the content of mineral elements, such as Ca, P, and K; of vitamin C; and of free amino acids; and also increased the total weight of the broccoli sprouts. The content of sulforaphane, a bioactive compound, also increased by up to 47% with illite treatment, with the highest increase being in the IPB-5 group. However, several of the parameters were lower in the IPB-7 group. Aromatic compounds were categorized by functional groups such as hydrocarbons which numbered 36, 30, 34, 28, and 30 in the control, IPB-1, IPB-3, IPB-5, and IPB-7 groups, respectively. We found 16, 15, 15, 13, and 14 sulfides, including dimethyl sulfide, in the control, IPB-1, IPB-3, IPB-5, and IPB-7 groups, respectively. Additionally, aldehydes, comprising seven compounds, were detected in the IPB-1, IPB-3, IPB-5, and IPB-7 groups. Illite treatment significantly increased the activities of antioxidants such as DPPH and the polyphenol content of the microgreens. These results indicate a potential role for appropriate illite doses in microgreen treatment to address multinutrient deficiencies and to increase the quality of microgreen vegetables.

## 1. Introduction

Microgreens have been extensively produced in recent decades and have been recognized as a potential source of nutrition [1]. The use of ‘microgreen’ seed sprouts as functional foods has become popular [2]. As such, improvements in the germination process could substantially enhance the nutritional values of sprouts [3]. The seed germination process not only transforms nutrients in seeds but also favors release of novel bioactive compounds [4].

Indoor culturing of microgreens in cities has gained popularity, and broccoli seeds have been popular among consumers. Broccoli (*Brassica oleracea* L. var. *italica*) is an edible plant [5] that belongs to the cruciferous family, is a rich source of vitamin C and K, and contains glucosinolate compounds and sulforaphane [6]. Broccoli sprouts are widely consumed raw to add crunch and texture to pasta, sandwiches, salads, and dressings [7,8].

Sulforaphane in broccoli has several health benefits for humans [9,10]. Sulforaphane obtained from glucoraphanin in broccoli sprouts has potent effects in humans such as anticancer effects; inhibiting angiogenesis; reducing inflammation, increasing the levels of detoxifying enzymes; protecting against brain conditions including Alzheimer’s, stroke, and Parkinson’s disease; and improvement in the symptoms of autism spectrum disorder [11,12,13].

Seed pretreatment to increase the quality, nutritional value, and yield of sprouts has been widely practiced. Efficient and safe tools must be selected for seed treatments to enhance seed quality, nutrition, yield, and safety [14,15]. We have found that seed treatment with illite notably increased germination, yield, and texture and improved the physiological properties of various sprouts such as brown rice, lettuce, and soybean [16,17]. Illite is a natural clay mineral that mainly contains potassium, calcium, iron, and magnesium. Few studies are available on the effect of illite treatment on microgreens. In this study, the aim was to investigate the effect of illite treatment on the quality characteristics, antioxidant activities, and bioactive compounds of broccoli sprouts. The findings provide new insight into the value of illite treatment in microgreen development and processing.

## 2. Results and Discussion

### 2.1. Effect of Soaking Time on Germination Rate of Broccoli Seeds

**Figure 1:**
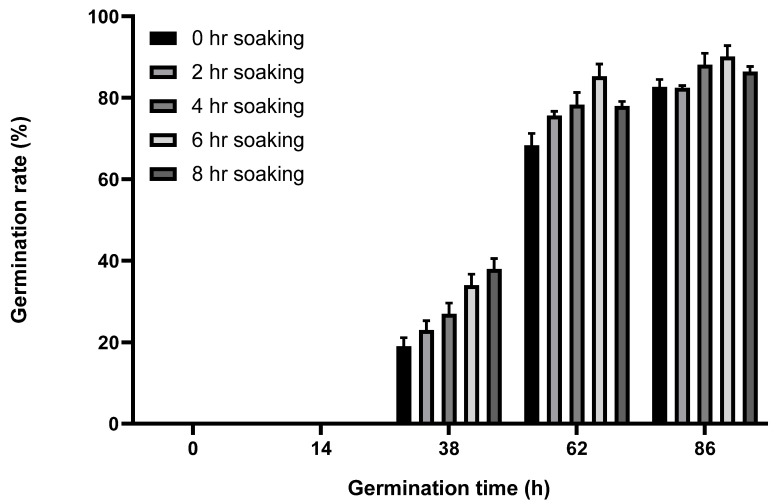
Germination rate (%) of broccoli seeds at different time intervals, grown after different soaking times.

Seed soaking is a crucial aspect of plant cultivation because soaking breaks seed dormancy and promotes germination. In this study, broccoli seeds were soaked under controlled conditions to promote germination. As shown in Figure 1, the optimum soaking duration of the broccoli seeds was determined by submitting them to soaking treatments at a fixed cultivation temperature (25 °C) and different soaking times, with germination rate (%) as the measured parameter.

No germination was observed in any of the treatment groups from 0 to 14 h after incubation. However, the germination rate sharply increased in all treatment groups after 14 h and up to 62 h, which was followed by gradual plateauing. All treatment groups exhibited germination rates exceeding 80% by the 86 h mark. The germination rates of the 0 and 2 h soaking treatment groups were 80% and 85%, respectively, at 86 h, whereas those of the 4, 6, and 8 h soaking treatment groups were 91%, 92%, and 90%, respectively, with no statistically significant differences among them. The germination rates of the 4, 6, and 8 h soaking treatment groups were 5–12% higher than those of the 0 and 2 h soaking treatment groups. These results are consistent with those of previous research, such as those of Park (2008) [18], showing that soaking for more than 4 h led to an increase in the germination rate of over 20% compared with that of an unsoaked control group.

The germination rate of the 6 h soaking treatment group was higher than the 4 and 8 h soaking treatment groups, indicating that a 6 h soaking duration is the optimal condition among those considered for promoting germination.

### 2.2. Total Weight and Vitamin C Content

Yield and vitamin C content of the broccoli sprouts were measured to evaluate quality characteristics after treatment with illite, and the results are presented in Table 1.

Broccoli sprouts are typically suitable for consumption when they are about 7 days old and are approximately 3–7 cm long. On the seventh day of illite treatment, the broccoli sprout yield was higher in all the illite treatment groups (ranging from 104.97 ± 4.81 g to 116.79 ± 1.89 g) than in the control group (101.44 ± 5.32 g). Notably, the yield of the IPB-1 treatment group (116.79 ± 1.89 g) was the highest, being approximately 15% higher than that of the control group (101.44 ± 5.32 g). However, the increase in illite treatment concentrations decreased the yield in the IPB5 and IPB-7 groups. Hence, the appropriate illite concentration to maximize yield must be determined.

Broccoli contains higher levels of vitamin C than lemons [19]. The ranking of vitamin C content in the treatment groups was as follows: IPB-3 (55.55 ± 3. 74 mg/100 g of dw) > IPB-1 (53.8 ± 3.17 mg/100 g of dw) > IPB-5 (50.5 ± 3.37 mg/100 g of dw) > IPB-7 (49.99 ± 1.32 mg/100 g of dw) > control (45.45 ± 1.75 mg/100 g of dw). All the illite-treated groups had considerably higher vitamin C contents than the control group.

Increases in vitamin C content with external treatment have been reported by various authors. Natella et al. [20] reported higher vitamin C contents in broccoli sprouts subjected to various elicitation treatments in sucrose-, mannitol-, and NaCl-treated groups than in a control group. Nishikawa et al. [21] reported similar results. In this study, the higher vitamin C content in the illite-treated groups (1%, 3%, and 5% treatments) compared to the control group might have been due to an appropriate balance of ions influx and efflux; however, osmotic disruption might have occurred with the highest illite treatment concentrations, which may have resulted in decreases in the vitamin C content in these treatments.

From our analysis, we found that the illite-treated solution contained a number of essential mineral elements (Table 2). Mineral elements play crucial roles in preserving and synthesizing several vitamin components. As such, we verified whether the treatment minerals influenced the nutrients, ions influx/efflux, yield, and vitamin C content of the sprouts. Our results agree with the findings of Salama et al. [22], who found that an organic and bio-organic fertilizer improved levels of antioxidants such as phenolics, flavonoids, and vitamin C in fennel cultivars. Uchendu et al. [23] reported the active role of vitamin C in alleviating lipid peroxidation in blackberries, preventing oxidative stress and improving cryopreservation. Kathi et al. [24] reported that mineral elements linked with vitamin C increased levels of essential nutrients in arugula microgreens. In addition, various biological factors such as light stress (which influences photo-oxidation), ionic imbalance caused by Na^+^ and Cl^−^, and storage time in plasma agriculture affect the germination process, which may also impact sprout quality and germination, as observed in mungbean, *Lepidium draba*, and radish sprouts [25,26].

### 2.3. Mineral Contents

Plants are autotrophic organisms capable of synthesizing nutrients essential for growth from basic inorganic elements [27]. Based on these phenomena, seed sprouts have been pretreated with several mineral elements and biological tools in practice to improve their nutritional properties. Care is required in application of treatment tools, as pathogens and toxic elements may result in food-borne illness and toxicity. In this study, the mineral contents of each of the illite water solution containing 1%, 3%, 5% and 7% illite were tested. It was observed that the Ca, K, Na, Mg, and Zn contents increased with the increasing illite concentrations (Table 2). 

**Table 2:** Quantification of mineral element (g/L) contents in illite-treated water solutions.

Element	Control	IS 1%	IS 3%	IS 5%	IS 7%
Ca	16.55 ± 0.34	22.15 ± 1.34	27.84 ± 3.4	34.42 ± 4.42	38.91 ± 2.37
Zn	0.01 ± 0.001	0.01 ± 0.0006	0.03 ± 0.0004	0.09 ± 0.016	0.10 ± 0.012
Fe	0.55 ± 0.02	0.89 ± 0.04	1.73 ± 0.08	2.58 ± 0.12	3.36 ± 0.16
K	6.75 ± 0.45	19.29 ± 4.5	25.88 ± 4.77	41.66 ± 2.26	53.37 ± 1.84
Na	17.01 ± 2.18	27.22 ± 1.65	36.53 ± 1.92	43.25 ± 5.56	46.71 ± 5.45
Mg	0.44 ± 0.14	0.66 ± 0.15	0.833 ± 0.15	0.55 ± 0.02	0.86 ± 0.02
Mn	ND	ND	ND	ND	ND
Cu	ND	ND	0.01	0.01	0.02
Cd	ND	ND	ND	ND	ND
As	ND	ND	ND	ND	ND
Hg	ND	ND	ND	ND	ND
Pb	ND	ND	ND	ND	ND
Total	41.33505	70.243195	92.8627078	122.5665369	143.3464516

Values are expressed as mean ± standard deviation of three replicates. Control: Water only; IS 1%: Water containing 1% illite; IS 3%: Water containing 3% illite; IS 5%: Water containing 5% illite; IS 5%: Water containing 5% illite; IS; Illite Solution; ND: Nondetectable.

Additionally, the mineral contents of the broccoli sprouts on the seventh day of cultivation under the different illite treatments were shown in Table 3. The total mineral content of the broccoli sprouts considerably increased with the increases in illite treatment concentration, especially for IPB-1 (28.24), IPB-3 (29.15), and IPB-5 (29.73), compared with that of the control (24.04). With the further increase in illite concentration, the total mineral content started declining from IPB-5 (29.73) to IPB-7 (24.98), which was considerably lower than that in the other treatments. Elements such as copper (Cu), iron (Fe), sodium (Na), zinc (Zn), and manganese (Mn) were detected in trace amounts in all treatment groups. These findings indicate that the IPB-3 illite concentration may be a maximum threshold for several of the minerals absorbed by broccoli microgreen sprouts. 

The calcium (Ca), potassium (K) ion, phosphorus, and sulfur contents were considerably elevated with the increases in illite concentration, except in the IPB-7 treatment, where the K and P contents were almost similar to those of the control (Table 3).

Interplay of Ca^+^, K^+^, P, and S has an important role in maintaining homeostasis in cells of plants, animals, and humans [28]. As such, fluctuations in content levels of ions such as K^+^/Na^+^ and Ca may adversely affect uptake of mineral ions. These elements are crucial elements for livestock and human health [29]. The contents of these ions in the broccoli microgreens, which increased with increasing illite concentrations up to the illite concentration of 5% in our experiment, could be considered normal. The diminishing attributes of the IPB-7 group demonstrated the potential negative effects of applying an illite concentration above 7%. However, the observed uptake of these elements signifies that deficiencies in macro- and micronutrients in food can be compensated for with illite treatment in the diets of humans and livestock.

### 2.4. Hunter’s Color Value

Color preference is a factor that strongly influences consumer choices regarding cooking, food safety, and food price, including esthetic values of food and products [30]. Color is also used as a standard for determining certain components, such as purple and orange for determining anthocyanin and beta-carotene contents, respectively [31]. Use of synthetic colors in food treatment may be unsafe due to harmful elements in these treatments that may result in various health ailments [32]. Processing of micronutrients contained in food, such as carotenoids retained during blanching, may result in undesirable colors and flavors through inactivation of peroxidases [33]. Therefore, color is an important parameter for assessing tools applied for pretreatment, biofortification, or priming of seeds. As shown in Table 4, the color of the broccoli sprouts was affected by the different concentrations of illite in the treatments.

L* (lightness) values ranged from 64.80 to 66.63, and no significant differences were observed between the control and treatment groups. However, a* (redness) and b* (yellowness) values tended to increase as the illite treatment concentration increased. Among the treatment groups, the a* (redness) values were highest in the IPB-5 and IPB-7 groups, at 1.10 ± 0.06 and 1.12 ± 0.06, respectively. The b* (yellowness) value was highest in the IPB-7 treatment group, at 20.29 ± 0.22.

### 2.5. Free Amino Acid Content

Amino acids are important components of diet, being required for human health, and are precursors of several antioxidants, phytohormones, enzymes, and hormones in crops [34]. Amino acids regulate several defense mechanisms and immune responses in plants to defend against various biotic and abiotic stresses [35,36]. The amino acid contents of the broccoli sprouts under the various illite treatments are presented in Table 5.

The total amino acid content ranged from 24 to 28.5 mg/g dry weight in the treatment groups, being higher in all the treatment groups than in the control (21.70 mg/g of dry weight). Notably, the total amino acid content was highest in the IPB-5 treatment group at 29.33 mg/g dry weight, being approximately 35% higher than in the control, followed by IPB-1, which was 31% higher than in the control. The essential amino acid content was highest in the IPB-1 treatment group at 11.35 mg/g dry weight, whereas the nonessential amino acid content was highest in the IPB-1 and IPB-5 groups, at approximately 12 mg/g dry weight for each.

The contents of other nonessential amino acids were higher in all the treatment groups (ranging from 5.30 to 6.13 mg/g dry weight) than in the control (5.28 mg/g of dry weight). Taurine, phosphoethanolamine, sarcosine, α-amino adipic acid, 3-methylhistidine, and carnosine were not detected. Essential amino acids are involved in DNA and protein methylation, gene expression, and glucosinolate biosynthesis, the precursor of which is sulforaphane [37,38]. Illite application might play a crucial role in regulating amino acids, thereby influencing sulforaphane synthesis in broccoli microgreens. 

### 2.6. Sulforaphane Content 

Sulforaphane (SFN) is a functional compound found in broccoli, known as an isothiocyanate (low-molecular-weight heterocummulene: R-N=C=S). SFN is found in cruciferous vegetables such as broccoli, cauliflower, kale, mustard, turnip, and cabbage [39]. SFN plays roles in regulating carcinogen metabolism enzymes, acting as an antimutagenic agent, and exhibiting powerful anticancer effects [39,40]. 

The sulforaphane contents in the broccoli sprouts on the seventh day of cultivation under the different illite treatments were as presented in Table 6. Illite treatment increased the sulforaphane contents in all the treatments. Increasing/decreasing patterns of sulforaphane content with treatments have been reported by several authors. Guo et al. [41] reported that applying a suitable NaCl concentration tended to increase contents of SFN and other bioactive compounds. Similarly, Li et al. [42] reported a rise in sulforaphane content in 8-day-old broccoli sprouts soaked in slightly acidic electrolyzed water with varying available chlorine concentrations. Hence, sulforaphane content, an indicator of the functional compounds in broccoli, requires further multidimensional research on treatments with natural substances, stress, and more.

### 2.7. Volatile Compounds

The presence and quantities of volatile compounds, which were determined by their retention times, are presented in Table S1.

Through separation and identification of the volatile aromatic components of the broccoli sprouts in the various illite treatments (based on CAS numbers), we found that the control group had a total of 70 compounds, whereas IPB-1, IPB-3, IPB-5, and IPB-7 had 65, 69, 66, and 64 compounds, respectively. The illite treatments slightly reduced the number of separated and identified aromatic components (ranging from 64 to 69) compared with the untreated control group.

We categorized the aromatic components based on their functional groups (Table S1), finding that hydrocarbonswere present in 36, 30, 34, 28, and 30 compounds in the control, IPB-1, IPB-3, IPB-5, and IPB-7 groups, respectively. A total of 16, 15, 15, 13, and 14 sulfides, including dimethyl sulfide, were identified in the control, IPB-1, IPB-3, IPB-5, and IPB-7 groups, respectively. Additionally, aldehydes, alcohols, ester, acid, ketone, and heterocyclic carbon were found, and a higher percentage of unknown compounds were found in all the treatment groups. 

Regarding the quantities of the aromatic components in the broccoli sprouts treated with illite, we observed that the dodecane (in the hydrocarbon group) content exhibited the following trend: IPB-1 (7.46%) > control (6.34%) > IPB-3 (5.94%) > IPB-5 (4.53%) > IPB-7 (3.39%). The 5-(methylthio)-pentanenitrile (in the sulfide group) content was as follows: control (19.17%) > IPB-3 (19.15%) > IPB-5 (18.27%) > IPB-15 (14.86%) > IPB-7 (13.49%). The ranking of the content of furfural, in the aldehyde group, was as follows: IPB-1 (1.12%) > control (1.09%) > IPB-3 (1.00%) > IPB-5 (0.98%) > IPB-7 (0.81%).

Erucin (1-isothiocyanato-4-methylsulfanyl butene), which is structurally related to sulforaphane and is a promising anticancer agent, was also detected [43]. In general, sulforaphane synthesis occurs through hydrolyzation of glucoraphanin. We also detected 5-(methylthio)- pentanenitrile (in the sulfides group), which is a breakdown product of glucosinolates in broccoli via enzymatic hydrolysis of glucoraphanin [44].

### 2.8. DPPH Radical Scavenging Activity, Total Flavonoid and Polyphenol Contents

Phytochemicals such as polyphenols and flavonoids, which are vital to plant physiology, are produced by plants to protect themselves from stressors such as ultraviolet radiation and reactive oxygen species [45]. In this study, the DPPH radical scavenging activity and total flavonoid and total polyphenol contents of the broccoli sprouts under the different illite treatment conditions were measured (Table 7).

The results regarding electron-donating ability toward 1,1-diphenyl-2-picrylhydrazyl (DPPH•) showed that the radical scavenging capacity of the plants in the IPB-1 treatment was highest at 82.09 ± 0.31%. The plants treated with illite concentrations ranging from 3% to 7% had weaker radical scavenging capacities. Although the radical scavenging capacity of the IPB-1 treatment group (82.09 ± 0.31%) was slightly higher than that of the untreated control (81.51 ± 0.16%), the remaining treatment groups (IPB-3, IPB-5, and IPB-7) exhibited significantly lower radical scavenging capacities in the range of 80.10% to 78.18%.

The total flavonoid content of the broccoli sprouts decreased as the illite treatment concentrations increased. The total flavonoid contents of the control (185.65 ± 5.25 mg/100 g) and the IPB-1 (186.00 ± 4.55 mg/100 g) treatment group were the highest, with no statistically significant differences between them.

The total polyphenol contents were significantly higher in all the treatment groups (74.35~74.41 mg GAE/100 g) than in the control group (74.25 ± 0.09 mg GAE/100 g), with no significant differences observed among the treatment groups.

In summary, the illite treatments had limited impact on increasing the contents of the polyphenolic compounds and flavonoids of the broccoli sprouts during the germination process. However, the IPB-1 plants had a higher DPPH radical scavenging activity. This finding aligns with the results of Zielińska-Dawidziak and Siger [46], who found that total phenolic content of bean sprouts soaked in 5~10 nm FeSO_4_ solution was higher (1.94~2.01 mg/g dry matter) than in 0 mm FeSO_4_-treated bean sprouts (2.10 ± 0.15 mg/g dry matter). Bean sprouts treated with 15~25 mm FeSO_4_ had the lowest content (1.78~1.68 mg/g dry matter). Antioxidant activity of those sprouts increased compared to the 0 to 10 mm FeSO_4_ treatments but significantly decreased with the 15~25 mm FeSO_4_ treatment. Furthermore, the results of the DPPH radical scavenging analyses demonstrated that the illite treatments at concentrations above 1% may have reduced the radical scavenging activities of the broccoli sprouts.

Precaution is required when testing effects of mineral ions, as accumulation of certain minerals in plant cells may inhibit antioxidant activities and enhance oxidative stress by generating free radicals [47]. Presences of heavy metals such as Cd, Pb, As, Ni, and Cr may damage cell membranes and nucleic acids, induce lipid peroxidation and protein degradation, and result in cell death [48,49]. Illite treatment can be considered safe for crops because it is devoid of toxic metals and did not produce an abrupt shift in antioxidant activities. 

## 3. Materials and Methods 

### 3.1. Materials and Samples

All chemicals, reagents, and solvents were of analytical grade and purchased from Sigma Chemical Co. (St. Louis, MO, USA). Water was treated with a system of thermal mantles (Isopad Isomantle, Borehamwood, Hertz, UK) and a Milli-Q water purification system (Millipore, Bedford, MA, USA). *Brassica oleracea* L. var. *italica* cv. Ultra, North Brunswick, NJ, USA, seeds were used for the experiment. Illite (natural mineral powder, Medexhealing, Yeoju, Seoul, Republic of Korea) was used for the treatments.

### 3.2. Broccoli Seed Germination Test 

Prior to illite treatment, the broccoli seeds were soaked for 0 h, 2 h, 4 h, 6 h, or 8 h in tap water to determine the ideal soaking duration. Seeds (1 g) were weighed in a beaker and washed thoroughly with distilled water. The percent germination of broccoli seeds was determined by placing rinsed seeds on filter paper (70 mm, Whatman #1, Whatman International, Maidstone, UK) in sterile Petri dishes (100 × 15 mm, Fisher Scientific, Hampton, NH, USA): 50 seeds per dish, 3 dishes per treatment combination. The filter paper was moistened with distilled water (10 mL), and seeds were germinated at 25 °C. Seeds were considered to have germinated when the radical protruded from the seed coat by at least 2 mm. Germination was checked at 14, 38, 62, and 86 h, and the total percent germination was recorded. Additional distilled water was added as necessary to keep the filter paper moist during germination.

### 3.3. Sprouting Method

The germination test results showed that the optimum germination rate was achieved after soaking the seeds for 6 h. Hence, the seeds were washed and soaked for 6 h in tap water and treated with the appropriate concentration of illite powder solution (1%, 3%, 5%, or 7% *w*/*v*) in each treatment at 25 °C. After soaking, rinsing with distilled water, and sterilizing for 1 min with 70% ethanol at room temperature, the seeds were grown in a sprouter (Saesooni, EK Co., Seoul, Republic of Korea) with 1 L of distilled water in an incubator (25 °C). The sprouts were cultivated for the first two days in water; then the appropriate illite solution was applied every second day, with distilled water applied on the alternating days. In total, the sprouts were treated with illite solution on three days and with distilled water on four days in alternating days in each treatment. The water was changed every day, ensuring the same volume was used. Sprouts were harvested when the sprouts reached the commercial size on the seventh day after sowing. After harvesting, sprouts were weighed and frozen at −80 °C, freeze-dried (Scanlaf model 110-4 PRO, LaboGene, Lillerød, Denmark), ground using a mill (Retsch ZM 200, Retsch, Haan, Germany), and kept in a desiccator until analysis.

### 3.4. Determination of Vitamin C Content

The vitamin C content was analyzed following the method prescribed by the AOAC [50]. In brief, 5 g of ground sample was suspended in 7.5 mL of 3% metaphosphoric acid and homogenized (AM-8, Nihonseike Kaisha, Tokyo, Japan). The extract was diluted with 12.5 mL of the acid solution, then filtered and titrated with 0.025% 2,6-dichloroindophenol. The oxidation of vitamin C and the color change were observed and quantified.

### 3.5. Color Measurement

The L* (lightness), a* (redness, + or greenness, −), and b* (yellowness, + or blueness, −) values of the extracts were determined using a Chroma Meter (CR-300; Minolta Corp., Osaka, Japan). A Minolta calibration plate (YCIE = 94.5, XCIE = 0.3160, ZCIE = 0.330) and a Hunter Lab standard plate (L* = 99.44, a* = −0.13, b* = −0.16) were used to standardize the instrument using a D65 illuminant.

### 3.6. Determination of Mineral Composition

The method described by Skujins [51] was followed to analyze the mineral content of the broccoli sprouts. For the extraction, nitric acid (15.0 mL) and sample extract (0.5 mL) were suspended, and an equal amount of distilled water was added to the mixture for dilution. The mineral concentrations were determined using an inductively coupled plasma atomic emission spectrometer (ICP AES, Varian Vista, Victoria, Australia).

### 3.7. Quantification of Free Amino Acid Content

The free amino acid content of the broccoli sprouts was quantified according to the protocol described by Je et al. [52]. One gram of powdered sample was hydrolyzed with 6 N HCl (10 mL) in a sealed vacuum ampoule at 110 °C for 24 h. HCl was separated from the hydrolyzed sample using a rotary evaporator, and the reaction mixture was prepared with 0.2 M sodium citrate buffer (pH 2.2). The mixture was passed through a Sep-Pak C18 cartridge (Waters Co., Milford, MA, USA), which was followed by filtration using a 0.22 μm membrane filter (Millipore, Billerica, MA, USA). The amino acid profile was determined with an automatic amino acid analyzer (Biochrom-20, Pharacia Biotech Co., Stockholm, Sweden). The samples were run twice, and the results are expressed as mg/g dry weight.

### 3.8. Extraction and Determination of Volatile Compounds (GC/MS)

The volatile compounds of the broccoli sprouts were detected following a previously described method [53]. In brief, the sample was extracted via the addition of 200 μL of 3-octanol (50 ng/mL), which was followed by the addition of sodium sulfate (5 g) in screw-capped vials. The vials were shaken in an autosampler (Combi PAL G6504-CTC CTC Analytics, Zwingen, Switzerland), and the volatile compounds were allowed to absorb at 4 °C for 20 min using solid-phase microextraction (SPME) fibers (50 μm DVB/CAR/PDMS Supelco, Bellefonte, PA, USA).

Each sample was extracted and injected into a gas chromatography/mass spectrometry system (GC/MS; Agilent 7890B GC, Agilent Technologies Inc., Santa Clara, CA, USA) coupled to an Agilent MSD 5977B quadruple mass spectrometer (Agilent Technologies Inc., Santa Clara, CA, USA).

One microliter of the sample was injected into a capillary column (DB-WAX, 60 m long, 0.25 μm i.d., 0.25 μm film thickness, Agilent Technologies, Middleburg, VA, USA). The compounds were identified using a W11N17 (Wiley11-Nist17, Wiley, Hoboken, NJ, USA; Mass Finder 3). The relative quantity of the chemical compounds in each sample is expressed as a percentage based on the peak area produced in the GC chromatogram.

### 3.9. Determination of Sulforaphane Content (LC/MS)

To quantify the sulforaphane contents, 1 g of powdered sample was diluted using 100 mL of 50% ethanol (conversion factor per gram = 100×) via a serial dilution process that follows shaking for 1.5 min, sonication for 20 min at 40 °C, and finally shaking for 1.5 min. After the extraction process, each sample solution was centrifuged at 15,000 rpm. Then 100 μL of the supernatant was collected and mixed with 900 μL of ethanol, followed by centrifuging at 15,000 rpm (Dilution factor = 10×). The diluted samples were filtered through a 0.22 μm PVDF membrane filter for LC-MS/MS analysis.

The final conversion factor per gram of sample powder = 1000 × [sulforaphane ng/g sample powder].

Sulforaphane was separated from the extracts and analyzed using an Agilent Poroshell 120, EC-C18 2.7 μm (100 × 2.1 mm) column maintained at 30 °C. The amounts of sulforaphane in the sample were qualified and quantified using the MS spectra from LC-MS/MS (Agilent 1290 Infinity II liquid chromatography and 6470 series triple quadruple tandem mass spectrometer).

### 3.10. Determination of Antioxidant Activities

Broccoli sprout extraction was performed using 100% methanol, and the extract tested for DPPH, polyphenol, and flavonoid contents using a microplate spectrophotometer (Multiskan GO, Thermo Fisher Scientific, Waltham, MA, USA). The absorbance values were measured at 517 nm following the method of Adhikari et al. [54] for DPPH and at 760 nm for polyphenol according to the Folin–Ciocalteau method. For flavonoid quantification, the method described by Mohdaly et al. [55] was followed, and the absorbance was measured at 510 nm. The total polyphenol contents are reported as gallic acid equivalents (μg GAE/mL extract). Quercetin was used to plot the standard curve, and the flavonoid content was measured as quercetin equivalents (μg QE/mL extract).

### 3.11. Statistical Analysis

All results are presented as mean ± standard deviation (SD). Analysis of variance was conducted using PROC GLM in SAS 9.4 (SAS Institute Inc., Cary, NC, USA). The significant differences among treatment means were identified with Student’s *t*-test at a 0.05 probability level. All reported values are averages of triplicate experiments unless otherwise mentioned.

## 4. Conclusions

The results of this study validate the effects of the illite treatments on increasing the total weight; the vitamin C, mineral, free amino acid, sulforaphane, and aroma compound contents; and the antioxidant activity of microgreen broccoli sprouts. In particular, soaking treatment with 1–5% illite is ideal for improving the nutritional properties and yield of broccoli sprouts according to these findings. However, the results may differ for different varieties and under different environmental conditions.

## Figures and Tables

**Table 1:** Effect of different illite treatment concentrations on the yield and vitamin C content of broccoli sprouts.

Sample	Total Weight (g)	Vitamin C Content (mg/100 g of dw)
Control	101.44 ± 5.32 ^b^	45.45 ± 1.75
IPB-1	116.79 ± 1.89 ^a^	53.8 ± 3.17
IPB-3	107.51 ± 9.50 ^ab^	55.55 ± 3.74
IPB-5	105.10 ± 7.16 ^b^	50.5 ± 3.37
IPB-7	104.97 ± 4.81 ^b^	49.99 ± 1.32

Control, broccoli seeds soaked in tap water for 6 h; IPB-1, broccoli seeds soaked in tap water containing 1% (*w*/*v*) illite powder for 6 h; IPB-3, broccoli seeds soaked in tap water containing 3% (*w*/*v*) illite powder for 6 h; IPB-5, broccoli seeds soaked in tap water containing 5% (*w*/*v*) illite powder for 6 h; IPB-7, broccoli seeds soaked in tap water containing 7% (*w*/*v*) illite powder for 6 h. Values are expressed as mean ± standard deviation of three replicates. Values followed by different letters in the same column indicate a significant difference (*p* < 0.05), Tukey’s test.

**Table 3:** Quantification of mineral element (g/L) contents in illite-treated broccoli sprouts.

Sample ^(1)^					
Element	Control	IPB-1	IPB-3	IPB-5	IPB-7
Ca	4.67 ± 0.1 ^d (2)^	5.48 ± 0.15 ^c^	5.9 ± 0.21 ^b^	6.26 ± 0.27 ^a^	5.53 ± 0.34 ^bc^
Cu	ND ^(3)^	0.01 ± 0.00 ^a^	0.01 ± 0.00 ^b^	0.01 ± 0.00 ^b^	ND
Fe	0.07 ± 0.00 ^b^	0.08 ± 0.00 ^a^	0.07 ± 0.00 ^b^	0.07 ± 0.00 ^c^	0.04 ± 0.00 ^d^
K	3.92 ± 0.19	4.56 ± 0.22	4.68 ± 0.3	4.99 ± 0.1	3.61 ± 0.15
Mg	2.22 ± 0.06 ^b^	2.32 ± 0.02 ^a^	2.35 ± 0.02 ^a^	2.24 ± 0.01 ^b^	1.78 ± 0.06 ^c^
Na	0.94 ± 0.00 ^d^	1.14 ± 0.02 ^b^	1.08 ± 0.01 ^c^	1.29 ± 0.01 ^a^	0.79 ± 0.01 ^e^
Zn	ND	ND	ND	ND	ND
P	5.03 ± 0.11	5.88 ± 0.064	6.05 ± 0.06	6.16 ± 0.14	4.87 ± 0.22
S	7.14 ± 0.17	8.76 ± 0.42	9.04 ± 0.44	8.69 ± 0.34	8.32 ± 0.4
Mn	0.01 ± 0.00 ^e^	0.01 ± 0.00 ^c^	0.02 ± 0.00 ^b^	0.02 ± 0.00 ^a^	0.01 ± 0.00 ^d^
Total	24.04	28.245	29.155	29.73	24.98

^(1)^ Samples are defined in Table 1. ^(2)^ Values are expressed as mean ± standard deviation of two replicates. Values followed by different letters in the same row are significantly different (*p* < 0.05, Tukey’s test). ^(3)^ ND: nondetectable.

**Table 4:** Hunter’s color values of broccoli sprouts treated with different concentrations of illite.

Sample ^(1)^	Color Value ^(2)^		
	L* (Lightness)	a* (Redness)	b* (Yellowness)
Control	65.85 ± 0.66 ^ab (3)^	0.96 ± 0.07 ^b^	18.83 ± 0.47 ^b^
IPB-1	65.32 ± 0.41 ^b^	0.95 ± 0.09 ^ab^	18.19 ± 0.16 ^c^
IPB-3	66.63 ± 0.81 ^a^	0.80 ± 0.10 ^b^	19.06 ± 0.37 ^b^
IPB-5	66.29 ± 0.69 ^ab^	1.10 ± 0.06 ^a^	19.42 ± 0.23 ^b^
IPB-7	64.80 ± 0.51 ^b^	1.12 ± 0.06 ^a^	20.29 ± 0.22 ^a^

^(1)^ Samples are defined in Table 1. ^(2)^ L*, lightness (100, white; 0, black); a*, redness (−, green; +, red); b*, yellowness (−, blue; +, yellow). ^(3)^ Values are expressed as mean ± standard deviation of three replicates. Values followed by different letters in the same column are significantly different (*p* < 0.05, Tukey’s test).

**Table 5:** Free amino acid composition (mg/g of dry weight) of broccoli sprouts treated with different concentrations of illite.

Sample ^(1)^					
Amino Acid	Control	IPB-1	IPB-3	IPB-5	IPB-7
Essential amino acids Threonine	0.96 ± 0.02 ^e (2)^	1.40 ± 0.02 ^a^	1.01 ± 0.02 ^d^	1.38 ± 0.02 ^b^	1.32 ± 0.02 ^c^
Valine	1.10 ± 0.02 ^d^	1.56 ± 0.02 ^a^	1.23 ± 0.03 ^c^	1.45 ± 0.02 ^b^	1.44 ± 0.02 ^b^
Methionine	0.64 ± 0.01 ^b^	0.51 ± 0.01 ^c^	0.67 ± 0.01 ^a^	0.50 ± 0.01 ^c^	0.53 ± 0.01 ^c^
Isoleucine	0.96 ± 0.01 ^d^	1.39 ± 0.02 ^a^	1.07 ± 0.02 ^c^	1.29 ± 0.02 ^b^	1.29 ± 0.02 ^b^
Leucine	1.63 ± 0.02 ^c^	2.04 ± 0.03 ^a^	1.65 ± 0.03 ^c^	1.98 ± 0.03 ^b^	1.95 ± 0.02 ^b^
Phenylalanine	1.04 ± 0.01 ^d^	1.35 ± 0.02 ^a^	1.05 ± 0.02 ^d^	1.32 ± 0.01 ^b^	1.28 ± 0.02 ^c^
Lysine	1.76 ± 0.03 ^d^	2.27 ± 0.03 ^a^	1.74 ± 0.03 ^d^	2.24 ± 0.03 ^b^	2.12 ± 0.02 ^c^
Histidine	0.64 ± 0.01 ^c^	0.83 ± 0.01 ^a^	0.64 ± 0.01 ^c^	0.82 ± 0.02 ^a^	0.78 ± 0.01 ^b^
Subtotal	8.73	11.35	9.06	10.98	10.72
Nonessential amino acids Aspartic acid	1.57 ± 0.02 ^c^	2.04 ± 0.02 ^a^	1.49 ± 0.03 ^d^	2.06 ± 0.02 ^a^	1.93 ± 0.01 ^b^
Serine	0.65 ± 0.01 ^c^	1.09 ± 0.02 ^a^	0.61 ± 0.01 ^d^	1.11 ± 0.01 ^a^	1.01 ± 0.02 ^b^
Glutamic acid	3.09 ± 0.03 ^d^	4.62 ± 0.04 ^b^	2.89 ± 0.03 ^e^	4.87 ± 0.03 ^a^	4.43 ± 0.03 ^c^
Glycine	0.41 ± 0.01 ^a^	0.38 ± 0.01 ^b^	0.39 ± 0.01 ^b^	0.39 ± 0.01 ^b^	0.38 ± 0.01 ^b^
Alanine	0.91 ± 0.01 ^c^	1.14 ± 0.02 ^a^	1.85 ± 0.01 ^d^	1.17 ± 0.02 ^a^	1.08 ± 0.01 ^b^
Tyrosine	0.62 ± 0.01 ^d^	0.91 ± 0.01 ^a^	0.67 ± 0.01 ^c^	0.84 ± 0.02 ^b^	0.82 ± 0.02 ^b^
Arginine	1.51 ± 0.03 ^c^	1.87 ± 0.02 ^a^	1.86 ± 0.02 ^c^	1.79 ± 0.02 ^b^	0.92 ± 0.01 ^d^
Proline	ND ^(3)^	ND	ND	ND	ND
Subtotal	8.74	12.04	8.42	12.22	11.45
Essential to nonessential amino acid ratio	0.99	0.94	1.08	0.90	0.94
Other amino acids Phosphoserine	0.21 ± 0.01 ^a^	0.18 ± 0.01 ^b^	0.21 ± 0.02 ^a^	0.17 ± 0.01 ^b^	0.18 ± 0.01 ^b^
Taurine	ND	ND	ND	ND	ND
Phosphoethanolamine	ND	ND	ND	ND	ND
Urea	0.39 ± 0.01 ^b^	0.30 ± 0.01 ^d^	0.47 ± 0.01 ^a^	0.34 ± 0.02 ^c^	0.36 ± 0.02 ^c^
Sarcosine	ND	ND	ND	ND	ND
α-Amino adipic acid	ND	0.01 ± 0.01 ^a^	ND	0.01 ± 0.01 ^a^	ND
Citrulline	2.22 ± 0.02 ^c^	2.35 ± 0.03 ^a^	2.21 ± 0.03 ^c^	2.36 ± 0.03 ^a^	2.27 ± 0.03 ^b^
α-Amino-n-butyric acid	0.01 ± 0.01 ^a^	ND	0.01 ± 0.01 ^a^	ND	ND
Cystine	0.41 ± 0.02 ^b^	0.47 ± 0.03 ^a^	0.43 ± 0.01 ^b^	0.47 ± 0.01 ^a^	0.48 ± 0.02 ^a^
Cystathionine	0.03 ± 0.01 ^a^	0.04 ± 0.01 ^a^	0.04 ± 0.01 ^a^	0.04 ± 0.01 ^a^	0.04 ± 0.01 ^a^
β-alanine	0.04 ± 0.01 ^b^	0.09 ± 0.01 ^a^	0.05 ± 0.01 ^b^	0.08 ± 0.01 ^a^	0.09 ± 0.01 ^a^
β-amino isobutyric acid	0.03 ± 0.01 ^a^	0.02 ± 0.01 ^a^	0.03 ± 0.01 ^a^	0.02 ± 0.01 ^a^	ND
γ-amino-n-butyric acid	0.06 ± 0.01 ^a^	0.06 ± 0.01 ^a^	0.05 ± 0.01 ^a^	0.06 ± 0.01 ^a^	0.06 ± 0.01 ^a^
Ethanolamine	0.04 ± 0.01 ^b^	0.06 ± 0.01 ^a^	0.05 ± 0.01 ^b^	0.07 ± 0.01 ^a^	0.06 ± 0.01 ^a^
Hydroxylysine	0.02 ± 0.01 ^a^	ND	ND	ND	ND
Ornithine	0.02 ± 0.01 ^b^	0.05 ± 0.01 ^a^	0.05 ± 0.01 ^a^	0.05 ± 0.01 ^a^	0.05 ± 0.01 ^a^
1-methylhistidine	0.02 ± 0.01 ^c^	0.32 ± 0.02 ^a^	0.02 ± 0.01 ^c^	0.32 ± 0.03 ^a^	0.26 ± 0.02 ^b^
3-methylhistidine	ND	ND	ND	ND	ND
Anserine	0.25 ± 0.02 ^a^	0.15 ± 0.02 ^b^	0.22 ± 0.02 ^a^	0.11 ± 0.01 ^c^	0.15 ± 0.01 ^b^
Carnosine	ND	ND	ND	ND	ND
Hydroxyproline	0.9 ± 0.03 ^e^	1.01 ± 0.03 ^de^	1.47 ± 0.03 ^c^	2.04 ± 0.02 ^a^	1.90 ± 0.02 ^b^
Sub-total	5.28	6.12	5.30	6.13	5.90
Total free amino acids	21.70	28.52	24.13	29.33	27.21

^(1)^ Samples are defined in Table 1. ^(2)^ Values are expressed as mean ± standard deviation of two replicates. Values followed by different letters in the same row are significantly different (*p* < 0.05, Tukey’s test). ^(3)^ ND: not detected.

**Table 6:** Sulforaphane content (μg/g of dry weight) of broccoli sprouts treated with different concentrations of illite.

Sample ^(1)^	Control	IPB-1	IPB-3	IPB-5	IPB-7
Sulforaphane (μg/g of DW)	15.23 ± 1.12.07 ^b (2)^	22.36 ± 0.87 ^a^	21.36 ± 1.09 ^a^	22.49 ± 0.24 ^a^	22.14 ± 0.63 ^a^

^(1)^ Samples are defined in Table 1. ^(2)^ Values are expressed as mean ± standard deviation of two replicates. Values followed by different letters in the same row are significantly different (*p* < 0.05, Tukey’s test).

**Table 7:** DPPH radical scavenging activities and total flavonoid and total polyphenol contents of broccoli sprouts treated with different concentrations of illite.

Sample ^(1)^	DPPH (% Inhibition)	Total Flavonoids (mg QE ^(2)^/100 g)	Total Polyphenols (mg GAE ^(3)^/100 g)
Control	81.51 ± 0.16 ^b (4)^	185.65 ± 5.25 ^a^	74.25 ± 0.09 ^b^
IPB-1	82.09 ± 0.31 ^a^	186.00 ± 4.55 ^a^	74.41 ± 0.07 ^a^
IPB-3	79.53 ± 0.55 ^c^	177.93 ± 1.31 ^b^	74.35 ± 0.02 ^a^
IPB-5	78.18 ± 0.71 ^d^	166.00 ± 8.20 ^c^	74.37 ± 0.12 ^a^
IPB-7	80.10 ± 0.65 ^c^	166.35 ± 3.47 ^c^	74.41 ± 0.12 ^a^

^(1)^ Samples are defined in Table 1. ^(2)^ Quercetin equivalent. ^(3)^ Gallic acid equivalent. ^(4)^ Values are expressed as mean ± standard deviation of three replicates. Values followed by different letters in the same column are significantly different (*p* < 0.05, Tukey’s test).

## Data Availability

The raw data supporting the conclusions of this article will be made available by the authors on request.

## Supplementary Materials

The following supporting information can be downloaded at: https://www.mdpi.com/article/10.3390/molecules29184347/s1, Supplementary Materials Table S1. Volatile compounds of broccoli sprouts soaked after different concentrations of illite treatment (unit: peak area %).
