# Supplementary material for: Effect of Illite Treatment on Quality Characteristics and Antioxidant Activity of Broccoli (Brassica oleracea L. var. italica) Sprouts
Source: Molecules. 2024 Sep 13;29(18):4347. doi: 10.3390/molecules29184347 (PMC11434328; doi:10.3390/molecules29184347)
Supplement: Supplementary file 1 [file molecules-29-04347-s001.zip › molecules-3124782-supplementary.pdf]

Table S1. Volatile compounds of broccoli sprouts soaked after different concentrations of illite treatment (unit :peak area %)

Compounds[1)]	Formula	RT[2)]	Sample[3)]				
			Con	IPB-1	IPB-3	IPB-5	IPB-7
**Hydrocarbon**							
**2-methylheptane**	**C_8H_{18}**	**5.2674**	**0.16**	**0.18**	**0.12**	**0.16**	**ND**
**Octane**	C_8H_{18}	5.7407	0.28	0.27	0.28	0.31	**0.29**
**2,4-Dimethylheptane**	C_9H_{20}	5.8742	0.79	0.88	0.67	0.97	**0.96**
**4-Methyloctane**	C_9H_{20}	6.7174	0.49	0.50	0.42	0.51	**ND**
**2,5-Dimethyltridecane**	$C_{15}H_{32}$	7.6129	ND	ND	ND	ND	**0.07**
**3-Ethylhexane**	C_8H_{18}	9.2169	ND	ND	ND	ND	**0.12**
**2,3,4-Trimethylheptane**	$C_{10}H_{22}$	9.3150	0.50	ND	ND	0.44	**ND**
**3-Methyloctane**	C_9H_{20}	9.3188	ND	0.38	ND	ND	**ND**
**5,6-Dimethylundecane**	$C_{13}H_{28}$	9.3203	ND	ND	0.61	ND	**ND**
**Decane**	$C_{10}H_{22}$	10.8858	4.81	4.90	4.63	5.31	**5.31**
**5-Methyldecane**	$C_{11}H_{24}$	11.1462	0.67	0.35	0.63	ND	**0.67**
**2,5-Dimethylnonane**	$C_{11}H_{24}$	11.1477	ND	ND	ND	0.36	**ND**
**2,6-Dimethylnonane**	$C_{11}H_{24}$	11.3173	0.76	ND	0.68	0.80	**ND**
**1,6-Dicyclohexylhexane**	$C_{18}H_{34}$	11.5559	0.10	ND	ND	ND	**ND**
**2-Methyleicosane**	$C_{21}H_{44}$	11.5726	ND	ND	0.21	ND	**ND**
**5-Ethyl-2-methyloctane**	$C_{11}H_{24}$	12.4153	ND	ND	ND	2.80	**2.53**
**tricyclo[4.1.0.02,7]hept-3-ene**	C_7H_8	12.6251	0.41	ND	ND	ND	**ND**
**2-(octyl)benzene**	$C_{15}H_{24}$	12.6282	ND	ND	ND	ND	**1.07**
**2-(octyl)benzene**	$C_{15}H_{24}$	12.6282	ND	ND	ND	ND	**1.07**
**2,4-Dimethyldecane**	$C_{12}H_{26}$	12.6461	ND	0.48	0.50	0.12	**ND**
**4-Ethylheptane**	C_9H_{20}	13.1652	ND	0.33	ND	0.35	**ND**
**2,3-Dimethylnonane**	$C_{11}H_{24}$	13.3593	0.41	ND	0.37	ND	**ND**

**4-Methyldecane**	$C_{11}H_{24}$	13.3571	ND	1.14	ND	0.46	**1.18**
**5-Ethyl-2-Methyl- Heptane,**	$C_{10}H_{22}$	14.7049	0.08	ND	ND	ND	**ND**
**11-(2,2-Dimethylpropyl) henicosane**	$C_{26}H_{54}$	14.7142	ND	0.08	ND	ND	**ND**
**2,2,3,4-Tetramethylpentane**	C_9H_{20}	14.7171	ND	ND	0.15	ND	**ND**
**4,7-Dimethylundecane**	$C_{13}H_{28}$	15.3503	0.32	ND	0.21	ND	**0.50**
**2,6,11-Trimethyldodecane**	$C_{15}H_{32}$	15.3516	ND	ND	0.28	0.34	**ND**
**2,6,10-Trimethyltridecane**	$C_{16}H_{34}$	15.6746	0.26	0.31	0.34	0.42	**0.43**
**Undecane**	$C_{11}H_{24}$	15.6826	ND	ND	0.20	0.25	**ND**
**2,6,6-Trimethyloctane**	$C_{11}H_{24}$	15.8480	0.11	ND	ND	ND	**ND**
**Ethylbenzene**	C_8H_{10}	17.9119	ND	ND	ND	0.55	**0.58**
**5-Methylundecane**	$C_{12}H_{26}$	18.5109	0.10	ND	ND	ND	**0.12**
**3,3-Dimethylhexane**	C_8H_{18}	18.8184	ND	0.20	0.39	ND	**0.25**
**2-methylundecane**	$C_{12}H_{26}$	18.9787	ND	ND	0.14	ND	**ND**
**3-methyldecane**	$C_{11}H_{24}$	18.9827	0.16	ND	ND	ND	**ND**
**2,2-dimethylhexane**	C_8H_{18}	18.9831	0.07	ND	ND	ND	**ND**
**4-Propylheptadecane**	$C_{20}H_{42}$	18.9835	ND	ND	ND	ND	**0.12**
**2,5-Dimethyltetradecane-**	$C_{16}H_{34}$	19.9506	0.12	ND	ND	ND	**0.09**
**5-Methyldodecane**	$C_{13}H_{28}$	19.9526	ND	0.13	ND	0.39	**0.07**
**2,3,3-Trimethylhexane**	C_9H_{20}	21.3522	0.07	ND	ND	ND	**ND**
**4,4-Dimethylheptane**	C_9H_{20}	21.3556	ND	0.10	ND	ND	**ND**
**7-Propyltridecane**	$C_{16}H_{34}$	21.3628	ND	ND	0.06	ND	**0.07**
**Dodecane**	$C_{12}H_{26}$	21.5750	6.34	7.46	5.94	4.53	**3.93**
**4-Methyldodecane**	$C_{13}H_{28}$	22.2592	0.28	0.42	0.32	0.41	**0.36**
**2- Cyclohexane,**	$C_{10}H_{20}$	22.6852	ND	0.08	ND	ND	**ND**
**1-iodohexadecane**	$C_{16}H_{33}I$	22.7277	0.13	0.74	0.08	0.25	**0.07**

**5,7-dimethylundecane**	$C_{13}H_{28}$	23.1397	0.19	ND	ND	0.22	**ND**
**3,3,5-trimethylheptane**	$C_{10}H_{22}$	23.5940	0.16	ND	ND	ND	**ND**
**1-Iodooctane**	$C_8H_{17}I$	23.5976	ND	ND	ND	0.19	**ND**
**5-Ethyltetracosane**	$C_{27}H_{56}$	24.0655	0.07	ND	ND	ND	**ND**
**2,3,7-Trimethyldecane**	$C_{13}H_{28}$	24.3045	0.45	ND	ND	0.51	**ND**
**4,6-Dimethyldodecane**	$C_{14}H_{30}$	24.4204	0.70	1.11	0.58	0.81	**0.86**
**2,3,3-Trimethylpentane**	C_8H_{18}	24.9415	0.19	0.21	ND	ND	**ND**
**4-Methylundecane**	$C_{12}H_{26}$	25.3214	ND	ND	0.07	ND	**ND**
**2,6,10,15-Tetramethyl heptadecane**	$C_{21}H_{44}$	25.7502	0.07	0.30	0.18	0.17	**0.15**
**5-Ethyl-5-propylundecane**	$C_{16}H_{34}$	27.6440	ND	ND	0.08	ND	**0.15**
**4,4-Dimethylundecane**	$C_{13}H_{28}$	27.6480	ND	0.15	ND	ND	**0.41**
**2,6,11-Trimethyldodecane**	$C_{15}H_{32}$	27.8249	ND	0.74	ND	ND	**ND**
**1-Iodododecane**	$C_{12}H_{25}I$	27.8295	0.30	ND	ND	ND	**ND**
**4-Iodo-2-methyl-1-butene**	C5H9I	29.5323	ND	ND	ND	0.07	**ND**
**2-Methyl-1-pentadecene**	$C_{16}H_{32}$	31.5579	ND	ND	ND	0.13	**ND**
**2-Ethyl-1,4-cyclohexadiene**	$C_{10}H_{14}$	32.1640	ND	0.20	0.16	ND	**ND**
**2,6,8-Trimethyldecane**	$C_{13}H_{28}$	34.3770	ND	ND	0.34	ND	**ND**
**Tetradecane**	$C_{14}H_{30}$	34.3771	0.42	0.49	ND	0.43	**0.39**
**2,2'-Dimethylbutane**	C9H20	34.6760	0.09	0.07	ND	ND	**ND**
**1,3-Bis(1,1-dimethylethyl)benzene**	C14H22	36.1022	0.86	0.95	0.79	0.94	**0.85**
**1,2,3,5-Tetramethylbenzene**	C10H14	36.4186	ND	0.41	ND	ND	**ND**
**1,2,4,5-Tetramethylbenzene**	C10H14	36.4225	ND	ND	0.31	ND	**0.25**
**1,6,6-Trimethyl-3- methylene-1,4-cyclohexadiene,**	$C_{10}H_{14}$	36.4259	0.66	ND	0.22	ND	**ND**
**1-Iodoheptane**	$C_7H_{15}I$	37.7159	ND	0.16	ND	ND	**0.08**

**2-Hexene, 3,5,5-trimethyl-**	C_9H_{18}	39.8710	ND	ND	0.09	ND	**0.09**	
**1-ethynyl-4- Benzene**	$C_8H_5F^3$	43.4806	ND	ND	0.12	ND	**ND**	
**Isopropylcyclobutane**	C_7H_{14}	44.3549	ND	ND	0.07	ND	ND	
**Pentylcyclopropane**	C_8H_{16}	44.3596	0.08	ND	ND	ND	ND	
**Sub-total**			**21.66**	**23.72**	**20.24**	**23.20**	**22.02**	

**Sulfide**							
**Borane-methyl sulfidecomplex**	$C_2H_6S{\cdot}BH_3$	5.2089	4.29	ND	4.28	4.32	**4.33**
**Methanethiol**	CH_4S	4.6771	0.12	0.12	0.08	0.09	**0.11**
**Carbon disulfide**	CS2	5.0406	2.62	2.49	2.23	2.38	**2.14**
**Dimethyl sulfide**	C_2H6_S	5.2161	ND	4.61	ND	ND	**ND**
**2-(Methylthio)acetaldehyde**	C_3H_6OS	25.2345	0.08	ND	ND	ND	**0.06**
**Thiocyanic acid, methyl**	C_2H_3NS	25.9022	0.23	0.22	0.21	0.19	**0.21**
**Allyl Isothiocyanate**	C_4H_5NS	31.7090	0.75	0.77	0.61	0.69	**0.60**
**Methional**	C_4H_8OS	37.6390	0.15	0.43	ND	ND	**ND**
**Thiazole, 2,4-dimethyl-**	C_5H_7NS	37.7381	ND	ND	0.15	ND	**ND**
**Dimethyl Sulfoxide**	C_2H_6OS	45.2685	0.56	0.56	0.57	0.49	**0.45**
**4- (methylthio)- Butanenitrile**	C_5H_9NS	57.0036	8.32	7.45	7.64	8.23	**6.30**
**1-Cyano-2,3-epithiopropane**	C_4H_5NS	59.3021	ND	0.06	0.09	ND	**0.08**
**4-(Methylthio)-1-butanol**	$C_5H_{12}OS$	60.1991	0.64	0.42	0.52	0.60	**0.54**
**5-(methylthio)- pentanenitrile**	$C_6H_{11}NS$	64.5823	19.17	14.86	19.15	18.27	**13.49**
**Allyl trisulfide**	$C_6H_{10}S_3$	65.0364	0.08	ND	ND	ND	**ND**
**1-isothiocyanato- Propane**	$C_5H_9NS_2$	67.1902	0.56	0.66	0.64	0.56	**0.39**
**[(2- methylpropyl)thio]- Benzene,**	$C_{10}H_{14}S$	68.1605	0.07	0.09	0.07	0.09	**0.07**

**Erucin**	$C_6H_{11}NS_2$	74.3109	1.37	1.48	1.70	0.23	**0.90**
**Episulfide isomer 2**	C_5H_7NOS	93.8058	0.45	0.27	0.44	0.73	**ND**
**Sub-total**			**39.46**	**34.49**	**38.38**	**36.87**	**29.67**

**Ester**							
**Methoxymethane**	C_2H_6O	8.7214	ND	ND	ND	ND	**0.13**
**Methyl 2-hydroxybenzoate**	$C_8H_8O_3$	56.4340	5.92	7.69	5.63	7.40	**6.14**
**Sub-total**			**5.92**	**7.69**	**5.63**	**7.40**	**6.27**

**Alcohol**							
**2-(1-Phenylethyl)phenol**	$C_{14}H_{14}O$	27.6475	0.07	ND	0.08	ND	**ND**
**3-Methyl-1-hexanol**	$C_7H_{16}O$	50.3518	ND	ND	ND	0.09	**ND**
**2-Amino-1,3-propanediol**	$C_3H_9NO_2$	51.1771	ND	ND	ND	0.63	**ND**
**Phenylmethanol**	C_7H_8O	61.9590	0.71	ND	ND	0.48	**ND**
**1,5-Hexadien-3-ol**	$C_6H_{10}O$	68.6556	2.21	2.04	1.62	2.24	**1.48**
**propane-1,2,3-triol**	$C_3H_8O_3$	82.6056	1.44	1.53	0.74	1.75	**1.05**
**Sub-total**			**4.43**	**3.57**	**2.44**	**5.19**	**2.53**

**Aldehyde**							
**2-Methylpropanal**	C_4H_8O	5.9411	0.57	0.52	0.55	0.52	**0.54**
**2-Methylbutanal**	$C_5H_{10}O$	7.9949	0.91	0.90	0.96	0.91	**0.91**
**3-Methylbutanal**	$C_5H_{10}O$	8.1135	0.52	0.55	0.53	0.48	**0.50**

**Pentanal**	$C_5H_{10}O$	10.1112	ND	ND	0.13	0.11	**0.14**
**(2E)-2-Heptenal**	$C_7H_{12}O$	29.3850	ND	0.23	0.27	0.28	**0.27**
**4,4- dimethyl-5-oxo Pentanenitrile,**	$C_7H_{11}N$	31.5335	0.08	ND	ND	ND	**ND**
**Nonanal**	$C_9H_{18}O$	33.9265	0.41	0.33	0.36	0.35	**0.39**
**2-Ethylhexanal**	$C_8H_{16}O$	37.7236	0.30	ND	ND	ND	**ND**
**2-Furaldehyde**	$C_5H_4O_2$	38.1814	1.09	1.12	1.00	0.98	**0.81**
**Sub-total**			**3.88**	**3.65**	**3.80**	**3.63**	**3.56**

**Acid**							
**Allyl ethyl Carbonic acid,**	$C_6H_{10}O_3$	10.1059	0.12	0.10	ND	ND	**ND**
**Isocyanic acid**	CHNO	27.8287	ND	ND	ND	0.17	**ND**
**Methoxy methyl ester-,Acetic acid**	$C_4H_8O_3$	43.4602	ND	0.62	ND	ND	**ND**
**Methyl 2-methylhexadecanoate**	$C_{18}H_{36}O_2$	80.1408	ND	ND	ND	0.19	**ND**
**Sub-total**			**0.12**	**0.72**	**0.00**	**0.36**	**0.00**

**Heterocyclic carbon**							
**1H-Pyrrole benzofuran**	C_4H_5N	20.1008	ND	ND	0.11	ND	**0.11**
**3-Methyl-2,3-dihydro-**	$C_9H_{10}O$	35.7372	ND	ND	ND	0.22	**ND**
**1-Methoxy-1,3-cyclohexadiene**	$C_7H_{10}O$	44.8337	ND	0.08	ND	ND	**0.09**

**Sub-total**			**0.00**	**0.08**	**0.11**	**0.22**	**0.20**

Ketone							
**3-Methyl-2,4-nonanedione**	$C_{10}H_{18}O_2$	15.0200	ND	0.23	ND	ND	**ND**
**Pent-3-en-2-one**	C_5H_8O	17.0541	ND	ND	ND	0.09	**ND**
**2,2-Dimethylhexan-3-one**	$C_8H_{16}O$	18.5087	ND	0.10	ND	ND	**ND**
**3-Hydroxy-2-butanone**	$C_4H_8O_2$	26.8875	ND	0.31	0.11	0.19	**0.08**
**6-Methylhept-5-en-2-one**	$C_8H_{14}O$	30.3285	ND	ND	ND	0.08	**0.10**
**3-Nonanone**	$C_9H_{18}O$	37.7214	ND	ND	0.39	0.43	**0.38**
**2-Hydroxy-3-pentanone**	$C_5H_{10}O_2$	39.8563	ND	0.39	ND	ND	**0.19**
**octa-3,5-dien-2-one**	$C_8H_{12}O$	41.7911	0.21	ND	0.13	ND	**0.27**
**Sub-total**			**0.21**	**1.03**	**0.63**	**0.79**	**1.02**

Other							
**N,N-dimethyl Methylamine**	C_3H_9N	4.2972	0.27	0.14	0.11	0.19	**0.26**
**Dimethylamine-D1**	C_2H_6DN	8.7267	0.26	0.16	1.54	1.93	**ND**
**7-Hexyloxepan-2-one**	$C_{12}H_{22}O_2$	9.2637	0.14	ND	0.08	ND	**ND**
**N-(2- hydroxyethyl)-Dodecanamide,**	$C_{14}H_{29}NO_2$	10.8885	ND	ND	ND	1.33	**ND**
**3-Butenenitrile**	C_4H_5N	20.1000	0.13	0.13	ND	ND	**ND**
**Methacrylonitrile**	C_4H_5N	20.1014	ND	ND	ND	0.16	**ND**
**4-Pentenenitrile**	C_5H_7N	25.9568	ND	ND	0.07	0.11	**ND**
**5-methyl- Hexanenitrile,**	$C_7H_{13}N$	31.5322	ND	ND	0.12	ND	**0.11**
**1-Butanamine**	$C_4H_{11}N$	36.6902	ND	0.07	ND	ND	**ND**
**Sub-total**			0.80	0.50	1.92	3.72	**0.37**
**Unknown[5)]**			23.52	24.55	26.85	18.62	**34.36**
**Total**			**100.00**	**100.00**	**100.00**	**100.00**	**100.00**

[1)] Compounds: For the characterization, the following database were used; W11N17 (Wiley11-Nist17, Wiley, Hoboken, NJ, USA).
[2)] RT: Retention time.
[3)] Samples are defined in Table 1.
[4)] ND: Not detected.
[5)] Unknown: Not detected in database.
